# Tobacco smoke and morphine alter peripheral and CNS inflammation following HIV infection in a humanized mouse model

**DOI:** 10.1038/s41598-020-70374-7

**Published:** 2020-08-19

**Authors:** William D. Cornwell, Uma Sriram, Alecia Seliga, Viviana Zuluaga-Ramirez, Sachin Gajghate, Slava Rom, Malika Winfield, Nathan A. Heldt, David Ambrose, Thomas J. Rogers, Yuri Persidsky

**Affiliations:** 1 Center for Inflammation, Translational and Clinical Lung Research, Lewis Katz School of Medicine, Temple University, Philadelphia, PA 19140 USA; 2 Department of Pathology and Laboratory Medicine, Lewis Katz School of Medicine, Temple University, Philadelphia, PA 19140 USA; 3 Center for Substance Abuse Research, Lewis Katz School of Medicine, Temple University, Philadelphia, PA 19140 USA

**Keywords:** Immunology, Microbiology, Neuroscience

## Abstract

Tobacco smoking is common in HIV-infected patients, and is prevalent among intravenous opiate abusers. Conversely, intravenous opiate abusers are more likely HIV-infected, and opiate abuse is associated with more severe neuroinflammation. Given the coincident use of tobacco smoking among HIV-infected intravenous drug users (IVDUs), we set out to study the effects of smoke exposure, chronic morphine administration, and HIV infection using the NSG humanized mouse model. Our results show that smoke, morphine, and the combination promotes the decline in CD4^+^ T cells in HIV-infected mice. Further, chronic morphine administration increases the numbers of circulating CD8^+^ T cells which express the inhibitory receptor PD-1, as well as the cytolytic proteins perforin and granzyme B in the infected mice. We also found that the combination of smoke and morphine inhibited the expression of IL-1α, IL-4 and IL-17A. Finally, the combination of smoke and morphine exposure induces microglial activation following infection, as well as in the absence of HIV infection. To our knowledge, this is the first report to assess the combined effects of smoke and chronic morphine exposure on the inflammation associated with HIV infection, and demonstrate that these two insults exert significant neuroinflammatory activity.

## Introduction

Tobacco smoking increases risks for multiple causes of illness and death^1^. Approximately 20% of adults in the United States are cigarette smokers, and smoking is the leading cause of preventable mortality and morbidity, resulting in more than 425,000 deaths annually^2^. People who use illicit drugs are known to have high rates of cigarette smoking. In a 24-year follow-up of narcotics addicts who were admitted to drug treatment, the death rates of smokers were four times that of non‐smokers^3^. Current estimates suggest that 75% of human immunodeficiency virus (HIV)-infected individuals smoke tobacco^4,5^. Opiate drug abuse is a major contributing factor to the global acquired immune deficiency syndrome (AIDS) epidemic, and it is likely that over a third of HIV infections in the US can be linked to intravenous drug abuse, and global estimates suggest that almost 20% of intravenous drug abusers are infected with HIV^6–8^.

Among individuals living with HIV, studies have found that smokers are at greater risk than nonsmokers to develop bacterial pneumonia, oral lesions and AIDS dementia complex^9^. This was particularly apparent in the era prior to the use of antiretroviral therapy^10^. A recent study reported by Khanna et al.^11^ has shown that tobacco smoke administration to rats results in substantial inflammation within the CNS. This response includes an upregulation of several cytokines including TNF-α, IL-17, TGF-β, and CCL2. The latter results support the notion that smoke induces inflammatory responses in the brain, consistent with the observation that the risk for HIV-associated neurodegeneration is greater in smokers, compared to non-smokers^9^.

A variety of basic, preclinical, and clinical studies have established that the immune response of opioid drug abusers is suppressed. Opioid agonists including morphine (an opiate with predominant μ-opioid agonist activity) inhibit both antibody and cellular immune responses in vivo and in vitro^12–14^. Moreover, it is well established that μ-opioid administration increases susceptibility to a number of infectious agents, including opportunistic infections associated with AIDS^14^.

Reports indicate that intravenous drug abuse increases susceptibility to the neurodegeneration associated with HIV infection. Bokhari et al.^15^ have shown that chronic morphine administration promotes the development of neurodegeneration following SIV infection. Further, intravenous drug users (IVDUs) have higher rates of HIV encephalopathy, and related neuroinflammation, compared to infected non-drug^16^. IVDUs exhibit more rapid neurologic progression, and tend to show more extensive macrophage/microglial cell activation within the CNS^17^. Morphine can induce neurodegeneration through a process which includes the deterioration of the blood–brain barrier (BBB)^18^. Moreover, morphine promotes the traffic of leukocytes into the brain from the peripheral blood leading to an increase in inflammation. The combination of HIV infection and opiate drug abuse may then result in a greater degree of neurodegeneration than would be observed with HIV infection or opiate use alone. The co-incident insults of HIV infection (particularly with the accumulation of HIV products into the brain interstitium), with a mu opioid agonist, appears to result in activation of astrocytes and microglia leading to greater pro-inflammatory and neurotoxic activity^19^.

Given the high incidence of HIV infection in intravenous opiate abusers, and the frequent use of tobacco by HIV-infected individuals, there is a clear need to understand the combined influences of tobacco smoke and morphine on HIV-associated disease. In this report, we have used NSG (NOD *scid* gamma, NOD-*scid* IL2Rg^null^, NOD-*scid* IL2Rgamma^null^) mutant mice reconstituted with human CD34^+^ hematopoietic cells^20,21^ (humanized mice—hereinafter referred to as NSG) to study the effects of tobacco smoke and morphine on immunomodulation in the periphery and the brain during chronic HIV infection. Our findings demonstrate that tobacco smoke and morphine increase peripheral and neural inflammation independently and within the context of HIV infection.

## Materials and methods

### Humanized mice

NSG (NOD *scid* gamma, NOD-*scid* IL2Rg^null^, NOD-*scid* IL2Rgamma^null^) mutant mice readily support engraftment of human CD34^+^ hematopoietic stem cells^22^ and reconstitution of human immune cells in about 14 weeks after engraftment. NSG mice were purchased from The Jackson Laboratory (Sacramento, CA USA) and maintained in microisolator cages in appropriate sterile conditions, which include autoclaved cages, bedding, nestlets, and igloos along with irradiated food and filtered water. We used NSG mice engrafted with cells from two donors for testing the effects of tobacco smoke and morphine on the inflammatory response to HIV infection (the Jackson Laboratories). Reconstitution was checked by submandibular blood collection upon arrival and assessment of human CD45 by flow cytometry (data not shown). All animal experiments were approved by the Temple University Institutional Animal Care and Use Committee and conducted in accordance with the Temple University guidelines, which are based on the National Institutes of Health (NIH) guide for care and use of laboratory animals and with the ARRIVE (Animal Research: Reporting In Vivo Experiments) guidelines (www.nc3rs.org.uk/arrive-guidelines).

### Smoke and morphine administration

*Cigarette smoke exposure:* Smoke from filtered 3R4F Cigarettes (University of Kentucky, Lexington, KY) was delivered using a TED-10 smoking system (Teaque Enterprises, Woodland, CA) using standard parameters (ISO 1991). Mice were exposed in whole body chambers (WBC) to mainstream smoke generated by 35 mL puffs of 2 s duration taken every 30 s and delivered to the WBCs with a bias flow of 3.7lpm room air. Total particulate matter (TPM) concentration within WBCs was measured weekly via 3R4F cigarette TPM documentation and confirmed gravimetrically from filters downstream of the WBC. An average TPM between 300 and 350 mg/m^3^ throughout the duration of cigarette smoke exposure was consistently measured by this method. In addition, pulse oximetry was determined following exposure to confirm adequate oxygenation. Mice were exposed to cigarette smoke for two hours, 5 days a week for a total of 8 weeks. Control mice not exposed to cigarette smoke were exposed to filtered room air in identical WBCs for the same time period. The smoke administration resulted in carboxyhemoglobin levels in whole blood ranging from 5.4 to 7.6% and mice exposed to filtered room air alone registered values less than 1.3% as measured immediately after final exposure. These values were within the range measured in humans who smoke 1–2 pack (20–40 cigarettes) per day.

**Figure 1:**
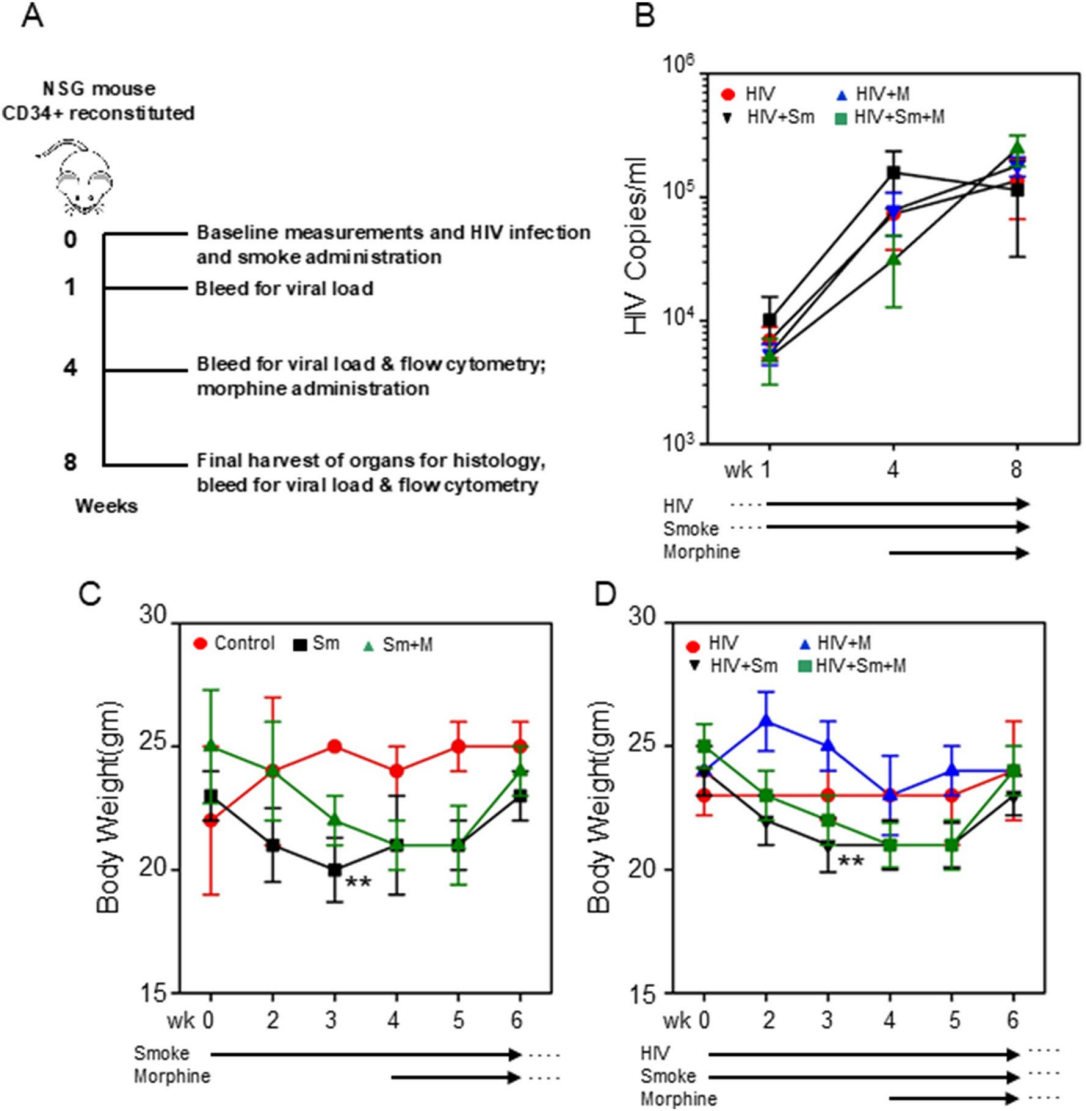
HIV viral load and body weight kinetics in smoke/morphine exposed NSG mice. (**A**) Experimental scheme. (**B**) Viral load (copies/mL) was analyzed in the plasma at the indicated time-points as described in the Methods section. Assessment of body weight in smoke/morphine exposed NSG mice (**C**), or smoke/morphine exposed and HIV-infected NSG mice (**D**). One-way ANOVA and unpaired t test statistics were used to compare the groups, as appropriate. **P < 0.01, comparing control vs. smoke and smoke + morphine groups (**C**); comparing HIV vs. HIV + smoke, and HIV + smoke + morphine groups (**D**).

### Morphine administration via mini-pumps

Morphine administration was conducted using 28 day Alzet mini-pumps. These pumps deliver a consistent and continuous dose of morphine (1 mg/kg/day) over a 28-day-period.

### HIV infection and viral load measurement in plasma

NSG mice from two donors were randomly assigned to the following treatment groups: control (n = 5), smoke (n = 8), smoke + morphine(n = 8), HIV(n = 7), HIV + smoke(n = 8), HIV + morphine (n = 8), HIV + smoke + morphine(n = 8). NSG mice were infected with the HIV_ADA_ strain at an MOI of 0.1 infectious particles/target cells in a volume of 50 µl per mouse. Some of HIV-infected mice were also exposed to tobacco smoke starting the same day of infection and morphine was administered via osmotic pumps 4 weeks later (Fig. 1A). A group of mice from each donor were not infected and used as controls. The morphine alone group did not survive the pump implants due to defective pumps, so we were unable to include that group in the analyses. Blood was collected at different time points (Fig. 1A) via sub-mandibular bleeding. Plasma viral load (Fig. 1B) was assessed by RealStar HIV RT-qPCR kit 1.0 (Altona Diagnostics, Hamburg, Germany) as per manufacturer’s instructions. PCR was run in a Step-One real-time PCR machine (Applied Biosystems, Foster City, CA) and HIV specific RNA copies were calculated from the standard curve.

### Flow cytometric analysis of peripheral blood T cell subsets

Blood was collected either by a cheek bleed (0 and 4 weeks) or a terminal bleed (8 weeks) into EDTA containing tubes to prevent coagulation. The whole blood was centrifuged at 300 g to pellet the cells and collect the plasma for storage. The pelleted blood was washed once with PBS followed by resuspension into PBS containing Fixable Viability Dye eFluor 455UV (ThermoFisher) and incubated for 30 min. The blood was washed with PBS and resuspended in FACS buffer (BD Bioscience) containing Fc Block (BD Bioscience) and incubated for 30 min on ice. The blood was centrifuged at 300 g for 10 min at 4 °C and the supernatant was removed. The blood was stained with a cocktail of anti-human antibodies including CD45-Alexa700 (BD Bioscience), CD3-BV510 (BioLegend), CD4-APC (eBioscience), CD8-eFluor450 (eBioscience), CD20-BUV395 (BD Bioscience), CD56-BUV395 (BD Bioscience), and PD1-PerCP-Cy5.5 (BioLegend) (Supplemental Table 1) in Brilliant Staining buffer (BD Bioscience) for 30 min on ice. The stained blood was washed once with FACS buffer (BD Bioscience) and the supernatant removed. The red blood cells were lysed with ACK Lysing buffer (ThermoFisher) for 10 min on ice followed by washing twice with FACS buffer. The cells were next fixed and permeabilized using eBioscience Transcription Factor Staining buffer kit (ThermoFisher) for 20 min on ice. The stained cells were washed once with 1X wash buffer in the kit. The intracellular antibody cocktail including Perforin-Pe-Cy7 (BioLegend) and Granzyme B-FITC (BioLegend) was added to the cells and incubated for 30 min on ice. The cells were washed once with FACS buffer and resuspended in FACS buffer. The samples were read on a BD LSRII flow cytometer and all events were collected. The data was analyzed using FlowJo (PC version 10.6; TreeStar) software. Compensation beads (BD Bioscience) were stained with each antibody individually to give single color controls and these were used to generate a compensation matrix in FlowJo which was applied to each sample. The gating strategy is presented in Supplemental Fig. 1).

### Measurement of plasma cytokines and chemokines

Multiplex ELISA kit for human cytokines and chemokines was purchased from MesoScale Discovery (Rockville, MD, USA; catalog No. K15054D1). This kit allows measurement of the following cytokines: Eotaxin, Eotaxin-3, GM-CSF, IFN-γ, IL-1α, IL-1β, IL-2, IL-4, IL-5, IL-6, IL-7, IL-10, IL-12/IL-23p40, IL-12p70, IL-13, IL-15, IL-16, IL-17A, IP-10, CCL2, CCL13, CCL22, CCL3, CCL4, CCL17, TNF-α, TNF-β, VEGF-A. Multiplex cytokine assay was performed on the plasma samples obtained from NSG mice at the 8-week time-point, as per manufacturer’s instructions. All samples were tested without dilution and all the cytokines (pg/ml) were detectable within the standard range provided by the manufacturer. Plates were read in an MSD ELISA plate reader and results were analyzed using GraphPad Prism software, version 7 (La Jolla, CA, USA).

### Immunohistochemistry

Microscopic examination of the NSG brains used a standardized protocol with sections from the neocortex (frontal and parietal), basal ganglia, and hippocampus. Paraffin Sections (5 μm) were stained with hematoxylin–eosin. One section from the frontal cortical lobe was used for the evaluation of neuroinflammation and BBB structure by immunohistochemistry using the following antibodies: IBA (1:100, Wako Chemicals USA, Richmond, VA, USA) for microglia activation. Primary antibodies were detected by Vectastain Elite Kit (Vector Laboratories, Burlingame, CA, USA) with diaminobenzidine substrate. Full sections (whole slide imaging) were captured using a 20 × objective by Aperio AT2 slide scanner (Leica Biosystems, Wetzlar, Germany). Immunostains for IBA-1 were assessed in blinded fashion using a semi-quantitative scoring protocol: 0—negative staining, 1—mild positivity (< 10% of cells), 2—moderate positivity (10–75%), and 3—strong positivity (75%)^23^. For each animal, 4–6 randomly selected fields in the cortex were analyzed. The values presented represent an average of these 4–6 fields.

### Statistical analysis

Results from all experiments were combined and expressed as mean ± standard error of mean (SEM). One-way ANOVA with Tukey’s multiple comparisons test or unpaired Student’s *t*-test was used as appropriate with *P* < 0.05 being significant.

## Results

### Progression of HIV infection during tobacco smoke and morphine exposure

NSG were infected with HIV_ADA_ and continued to be exposed to tobacco smoke (starting the same day of HIV infection) and morphine (starting four weeks after HIV infection) and analyzed for viral load at 4 and 8 weeks post infection. HIV copy numbers increased significantly in all the groups at 4 and 8 weeks (Fig. 1B). Neither smoke nor morphine individually or in combination increased the viral titer. Viral titers were elevated in smoke exposed HIV-infected mice than unexposed control mice (room air exposed) mice at 4 weeks, but did not reach statistical significance.

### Decrease in body weight upon smoke exposure in the presence or absence of HIV infection

Weights of NSG mice were assessed every week and results are presented for up to six weeks for all the study groups. There was a significant steady weight loss up to five weeks in mice exposed to tobacco smoke alone or in combination with morphine, as compared to control mice (Fig. 1C). While the baseline weights for these mice were much higher than the control mice, weight loss with smoke exposure alone was much higher than in combination with morphine, suggesting that tobacco smoke is probably responsible for this effect. A similar trend in weight reduction was observed in the HIV-infected mice groups exposed to smoke alone, morphine alone or smoke with morphine (Fig. 1D). Despite HIV infection, morphine did not cause significant body weight reduction. HIV-infected mice, without smoke or morphine exposure, also had steady weights until the end of the study. There was a gradual weight decrease in the initial five weeks for smoke alone and smoke in combination with morphine groups with or without HIV infection, but a weight rebound occurred by six weeks (Fig. 1D).

### Decrease in CD4/CD8 ratios in HIV-infected mice exposed to tobacco smoke and morphine

**Figure 2:**
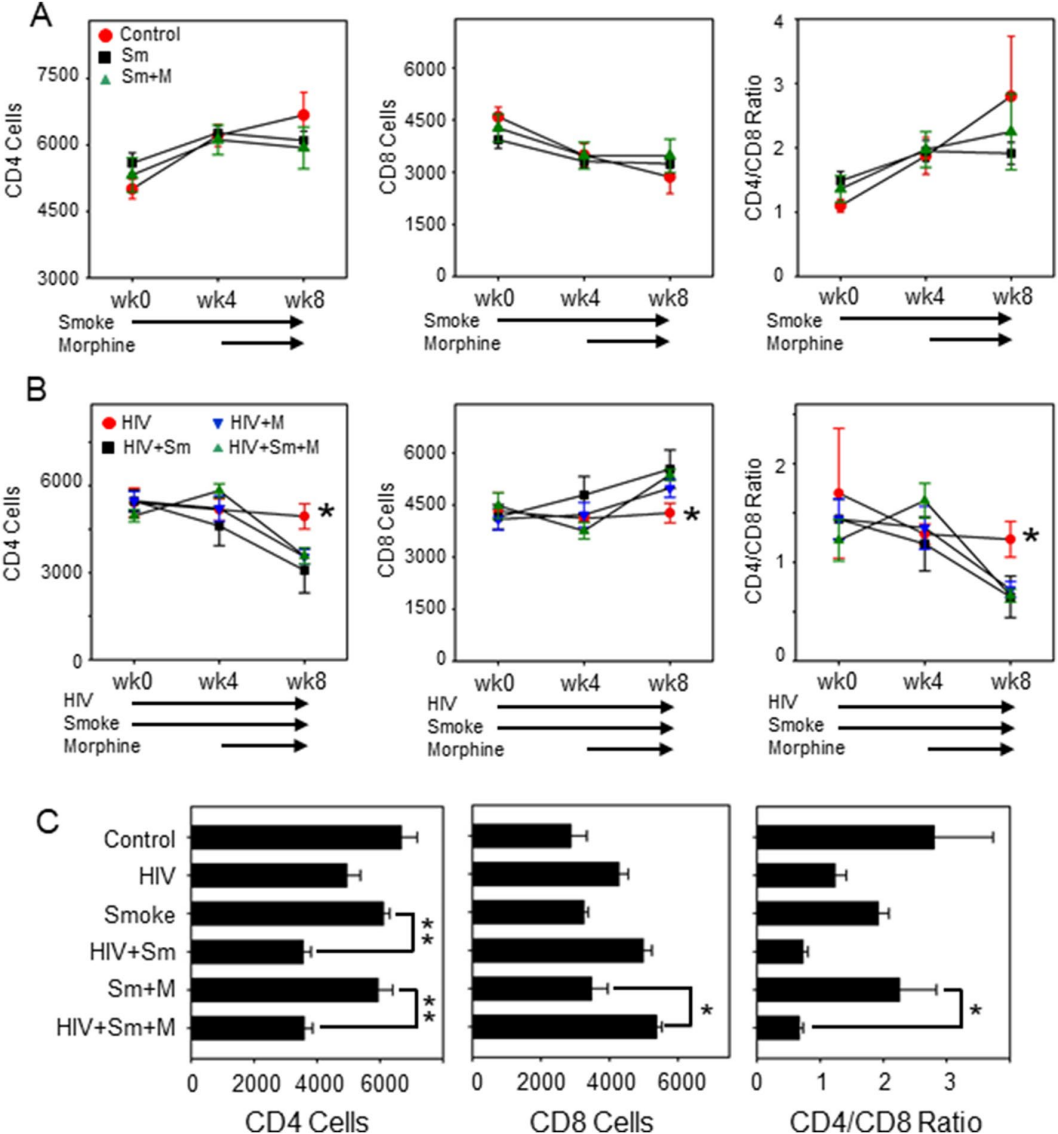
Peripheral blood T cell profile in NSG mice exposed to smoke/morphine and infected with HIV. Time-kinetics of CD4 and CD8 levels in the smoke and/or morphine exposed NSG mice (**A**) and HIV-infected NSG mice receiving smoke and/or morphine (**B**). Levels of CD4, CD8, or the ratio of CD4/CD8 T cells are presented (**C**). One-way ANOVA and unpaired t test statistics were used to compare the groups, as appropriate. *P < 0.05, **P < 0.01.

NSG mice were bled at baseline, 4 and 8 weeks post HIV infection, and the human T cells were analyzed for expression of CD3, CD4 and CD8. Results show that exposure to smoke and morphine, either alone or as a combined exposure, did not significantly alter absolute CD4 and CD8 counts or CD4/CD8 ratios compared to controls (Fig. 2A) in the absence of HIV. There was a significant reduction in CD4 counts, an increase in CD8 counts, and a decrease of CD4/CD8 ratios by 8 weeks in HIV-infected mice exposed to smoke and morphine (either alone or combined exposure) (Fig. 2B). Further analysis of CD4 and CD8 counts and ratios in smoke-exposed HIV-infected groups (smoke vs. HIV + smoke) and smoke combined with morphine exposure group (smoke + morphine vs. HIV + smoke + morphine) at 8-week time-point, revealed much greater reduction of CD4 counts and CD4/CD8 ratios, indicating that HIV infection in the background of smoke and morphine exposure worsened the CD4 T cell decline (Fig. 2C).

### Alterations in PD-1, perforin and granzyme B expression by T cells in NSG mice following smoke and/or morphine exposure and HIV infection

**Figure 3:**
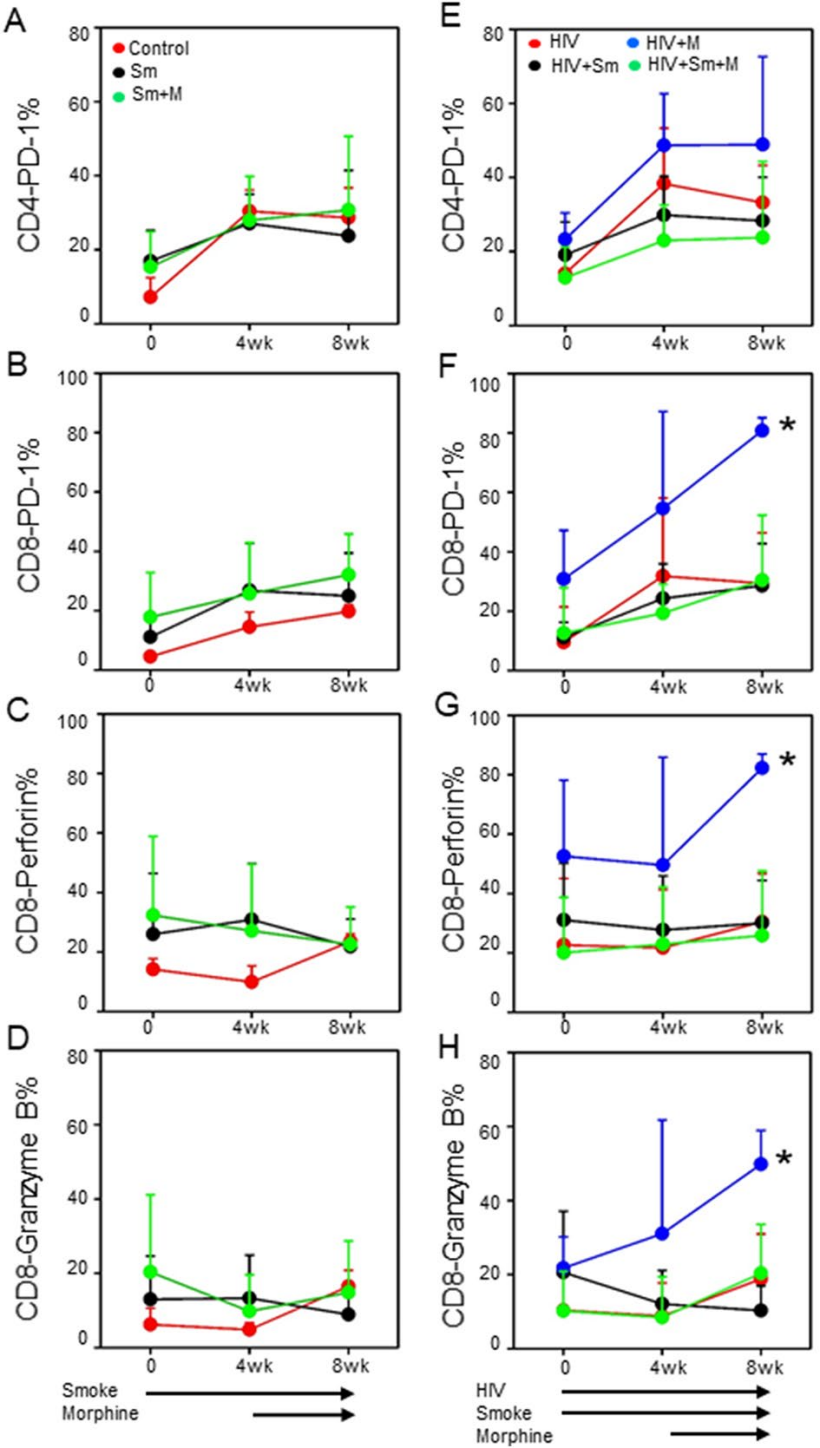
Expression of PD-1, perforin and granzyme B in T cells. Expression of PD-1 (in CD4 and CD8 T cells) and perforin or granzyme B (in CD8 T cells) in the smoke and morphine-treated NSG mice (**A–D**) and smoke and/or morphine exposed HIV–infected NSG mice (**E–H**); One-way ANOVA and unpaired t test statistics were used to compare the groups, as appropriate. *P < 0.05; **P < 0.01.

T cell exhaustion is a hallmark in chronic HIV infection highlighted by the increased expression of immune checkpoint markers such as PD-1^24^. Many studies in patients have demonstrated a clear correlation between increased PD-1 expression (on CD4^+^ and CD8^+^ T cells) and clinical outcome, such as accelerated decline of CD4^+^ T cells in acute and chronic infection^25,26^. Our results show that PD-1 expression by either CD4 or CD8 cells was not significantly altered by either smoke or the combination of smoke and morphine administration in the absence of HIV infection (Fig. 3A,B). We also found that PD-1 expression was not significantly altered by either smoke, morphine or the combination of smoke and morphine with HIV-infected CD4 cells (Fig. 3E). However, we found that PD-1 expression by CD8 cells following morphine administration to HIV-infected animals was significantly increased relative to any of the other HIV-infected treatment groups (Fig. 3F).

We also assessed the impact of morphine and smoke on the expression of perforin and granzyme B, two critical components of the anti-viral response to HIV. The results show that exposure to either smoke or the combination of smoke and morphine did not exert a significant effect on either perforin or granzyme B in CD8 T cells of uninfected animals (Fig. 3C,D). Moreover, the expression of perforin or granzyme B was not altered by either smoke or the combination of smoke and morphine in CD8 T cells from HIV-infected animals (Fig. 3G,H). In contrast, the expression of perforin and granzyme B by CD8 T cells was significantly up-regulated following morphine administration by CD8 T cells from HIV-infected mice (Fig. 3G,H).

### Modulation of the systemic cytokine/chemokine profile in NSG mice exposed to smoke and morphine with or without HIV infection

**Figure 4:**
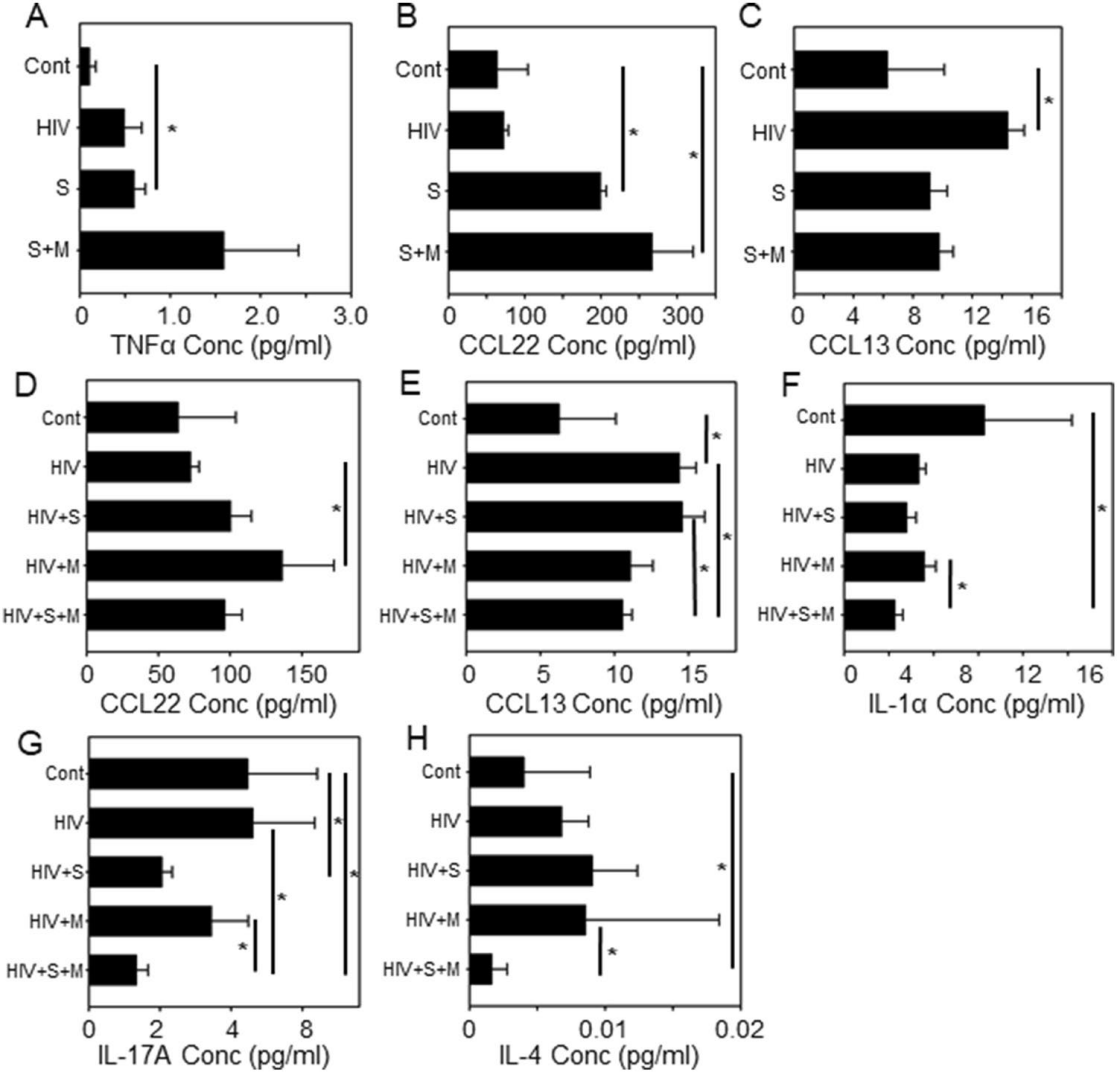
Human cytokine levels in animals exposed to either smoke or morphine in the plasma of NSG mice (**A–C**). Human cytokine levels present in the plasma of smoke and/or morphine- treated HIV-infected NSG mice (**D–H**). One-way ANOVA and unpaired t test statistics were used to compare the groups, as appropriate. *P < 0.05. The values are represented as fold change in log_2_ scale.

Cytokines and chemokines play an important role in regulating the immune responses to HIV infection, and exposure to smoke and morphine is known to alter the expression of these proteins^27–29^. First, we analyzed a panel of twenty-nine human chemokines and cytokines and compared the changes in cytokine and chemokine levels in the uninfected NSG mice exposed to smoke alone or co-exposed with morphine. The results showed that in the absence of HIV infection, the expression of TNFα was significantly elevated in the blood following smoke exposure, and the expression of CCL22 was significantly elevated following exposure to either smoke or the combination of smoke and morphine (Fig. 4A,B). Moreover, we also found that the expression of CCL13 was elevated by HIV infection, in the absence of either smoke or morphine expression (Fig. 4C). We found that the administration of morphine up-regulated the level of CCL22 in HIV-infected animals (relative to HIV alone) (Fig. 4D). In contrast, the level of CCL13 was significantly reduced by exposure to the combination of smoke and morphine in the HIV-infected animals, while the exposure to smoke alone or morphine alone did not have a significant impact on the level of this chemokine (Fig. 4E). The level of IL-17A was also reduced following exposure to the combination of smoke and morphine (relative to the HIV-infected but untreated animals) (Fig. 4G). Finally, the levels of IL-17A, IL-4, and IL-1α in HIV-infected animals exposed to the combination of smoke and morphine were all reduced relative to control (non-infected animals) (Fig. 4F–H).

An examination of the overall trends for the levels of 29 cytokines shows that exposure to smoke and the administration of both smoke and morphine were very similar (Supplemental Fig. 2). In this case, the same group of 10 cytokines that were up-regulated by smoke treatment, were also elevated by the combination of smoke and morphine. Also, the same 10 cytokines which were down-regulated by smoke treatment, were also down-regulated by the combination of smoke and morphine. For animals that were infected with HIV, the exposure to smoke alone, the treatment to morphine alone, and the treatment to the combination of smoke and morphine exhibited very similar changes in the levels of cytokines (Supplemental Fig. 3). Finally, in an effort to address the influence of the HIV infection on the levels of circulating cytokines, we first compared the levels of cytokines in control animals with the HIV-infected mice. The results show (Supplemental Fig. 4) that 10 cytokines exhibit at least a two-fold up-regulation, and 10 cytokines show at least a two-fold reduction relative to the control animals. A comparison of animals exposed to smoke alone with HIV-infected animals exposed to smoke showed a smaller number of cytokines with a two-fold increase or decrease with in the levels of cytokines. Finally, a comparison of animals treated with the combination of smoke and morphine, and HIV-infected animals treated with the combination showed that the levels of 26 of the 29 cytokines were reduced. This striking down-regulation in the HIV-infected animals exceeded a two-fold threshold for 19 of the 29 cytokines.

### Exposure to smoke and morphine increased neuroinflammation during HIV infection

**Figure 5:**
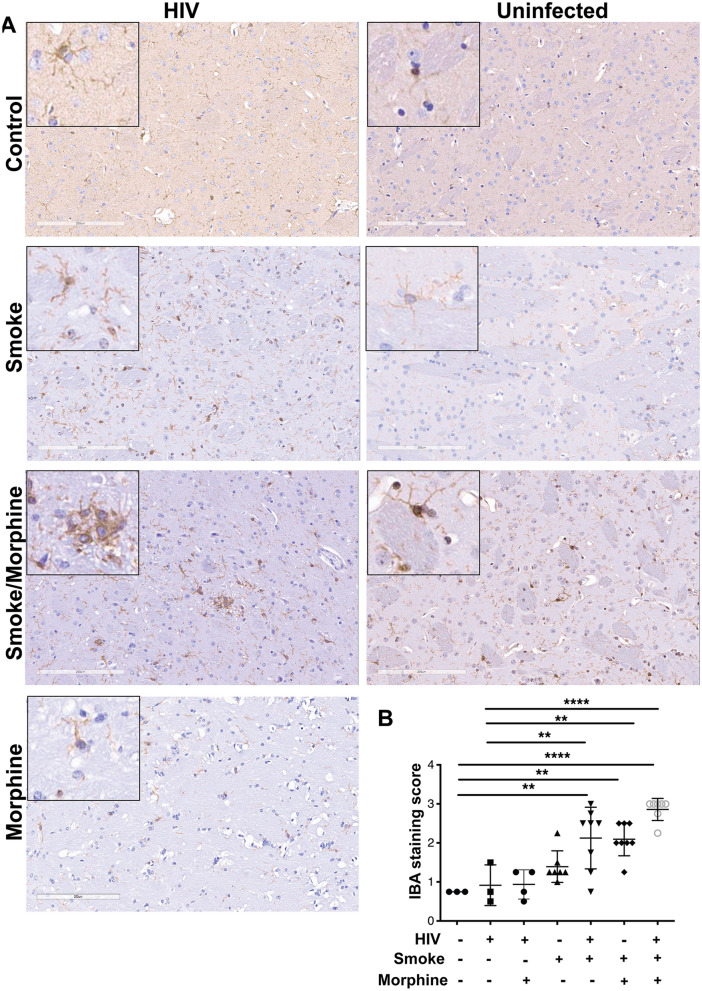
Microglial activation in HIV-infected NSG mice exposed to morphine, smoke or a combination of HIV, morphine and smoke. (**A**) Representative images of IBA-1 in basal ganglia in different animal groups. Whole slide scanning was performed and blindly evaluated. Original magnification × 200, inserts × 600. (**B**) Semin-quantitative assessment of microglia reaction in basal ganglia.

We analyzed neuroinflammation in the NSG cohort by immunohistochemistry. Microglial activation was analyzed by staining for IBA-1. HIV infection alone did not increase IBA-1 expression significantly (Fig. 5A,B). Moreover, treatment with morphine in the presence of HIV infection did not induce a significant level of microglial activation. While smoke without HIV infection failed to significantly enhance IBA-1 expression, HIV-infected animals exposed to smoke showed a significant augmentation of microglial activation (Fig. 5A,B). Both uninfected and HIV-infected animals treated with the combination of smoke and morphine exhibited a significant increase in the level of microglial activation. This was particularly notable for the uninfected animals that received the combination of morphine and smoke.

## Discussion

In addition to the high level of coincident tobacco use among intravenous drug abusers, evidence suggests that a majority of heroin abusers are also tobacco smokers, and the smoking lengthens the duration of heroin reinforcement^30^. Furthermore, clinical data indicate that over 85% of methadone-supported patients use tobacco products^31^. Given this background, we set out to examine the combined effects of chronic opioid administration and tobacco smoke on the immunopathogenesis of HIV infection in a humanized mouse model. As far as we know, this is the first study to examine the combined effects of chronic opioid administration and tobacco exposure on the inflammatory response to HIV infection. Our results show that tobacco smoke exposure, chronic morphine administration, and the HIV infection each have diverse effects on the immune system. For example, we observed that the smoke and/or chronic opioid administration, or the combination, promoted the decline in circulating CD4 counts, and increased the numbers of CD8 counts, in HIV-infected animals. Previous literature has been somewhat inconsistent in terms of the impact of either chronic opioid or smoke administration on the levels of CD4 T cells.

Heroin addicts have been reported to exhibit reduced numbers of CD4 T cells in the blood, and a coinciding reduction in CD4/CD8 ratio in some studies^32,33^, while opposing results from both clinical and pre-clinical studies have been reported by others^34,35^. The inability to observe more uniform results may reflect differences in the nature of the drug abuse in the clinical studies (e.g., multi-drug use) and the variance in the experimental design in the non-human primate studies^35^. However, in the absence of HIV infection, our data show that opioid exposure failed to alter the levels of CD4 or CD8 cells. These results are consistent with our previous studies which showed that chronic morphine administration of morphine to macaques had no effect on the circulating levels of either CD4 or CD8 cells^36^.

There is disagreement regarding the effect of tobacco smoking on the rate of decline of CD4 counts among HIV-infected patients^10^. Moreover, there is also a divergence of data regarding the impact of smoking on time to progression to AIDS, as well as AIDS-related mortality. However, for patients receiving antiretroviral therapy, there appears to be no significant association of smoking with mortality^10^. Nevertheless, the present results are consistent with previous reports which have shown an accelerated decline in blood CD4 numbers, particularly among patients who are heavy smokers^10,37^. It should be pointed out that these studies are in contrast to reports which show an increase in circulating CD4 and CD8 counts in smokers in the absence of HIV infection^38,39^, in contrast to our data reported herein. Nevertheless, in the present studies we observed a significant change in circulating CD4 and CD8 levels following either smoke exposure, chronic morphine administration, or the combination of these insults with HIV infection. These results were somewhat surprising given our data showing that the smoke and opioid administration failed to alter the blood levels of HIV.

We assessed the phenotype of circulating T cells in infected and non-infected animals, exposed to either smoke or chronic morphine, or the combination. The results showed that neither smoke nor morphine exposure altered the expression of the T cell inhibitory receptor PD-1 in non-infected animals. In HIV-infected animals, the expression of PD-1 by CD8^+^ T cells increased substantially in the animals receiving chronic morphine administration. On the other hand, smoke exposure alone, and the combination of smoke and morphine, failed to increase the expression of PD-1. The expression of PD-1 has been referred to as the “master” off-signal to down-regulate the immune response following a round of immune activation^40^. However, when antigen stimulation persists, as in the case of a chronic viral infection, T cells can undergo partial maturation and up-regulate PD-1 expression. For CD8^+^ T cells, this maturational stage is otherwise known as T cell exhaustion, these cells fail to manifest normal antiviral activity in part because of a reduction in the expression of normal levels of cytokines or the production of normal levels of cytolytic granule components^40,41^. We were surprised to find that animals receiving chronic morphine administration exhibited significantly greater numbers of perforin and granzyme B expressing CD8^+^ T cells. Here again, the up-regulation of perforin and granzyme B was not apparent in the animals receiving either smoke alone or the combination of smoke and morphine. It is not clear why the chronic morphine administration induced both an up-regulation of PD-1 as well as increased granule components. However, it appears that T cells in the “exhaustion” stage of partial maturation can be induced to progress to a more mature stage of maturation and exhibit elevated levels of both PD-1 and the granule proteins^40–42^. The regulation of inhibitor receptor expression, such as PD-1, as well as the granule components perforin and granzyme B, is known to be under the control of a set of transcription factors including Tim-3, Eomes, and T-bet^43,44^. Little is known regarding the regulation of gene expression in T cells following chronic opioid administration, but it is possible that the morphine treatment may alter the level of expression of these transcription factors.

Infection with HIV results in an up-regulation of a number of pro-inflammatory cytokines, including IL-1, IL-6, CCL2 and TNFα^45,46^. However, in the present report we analyzed the levels of 29 cytokines and other biomarkers, and we found that only CCL13 was significantly elevated following HIV infection. Of course, our data are derived from NSG mice, and are limited to an assessment of the levels of human cytokines. In the NSG mice, the source of cytokines would be limited to the grafted lymphocytes and myeloid cells. We also observed that exposure to smoke induced elevated the level of TNFα, and both smoke and the combination of smoke and chronic morphine up-regulated the level of CCL22 in non-infected animals. The observation of smoke induced TNFα is consistent with previous studies of the effects of smoke on the inflammatory response^47,48^. Moreover, we observed that chronic morphine administration elevated the level of CCL22 in the context of HIV infection. However, we found that the combination of smoke and morphine administration induced a significant reduction in the levels of IL-1α, IL-4 and IL-17A, while neither smoke nor morphine alone mediated the same inhibitory effect. These results would be consistent with the general observation that morphine is immunosuppressive^12,49,50^, but we did not observe the same effects on cytokine levels with the HIV-infected animals receiving morphine alone. On the other hand, in general, smoke exposure has been reported to result in elevated levels of several pro-inflammatory cytokines, including IL-1β, IL-6 and TNFα as noted above. However, there are components of smoke that have immunosuppressive activity, including nicotine, and these components may when combined with morphine may be responsible for the inhibition of IL-1α, IL-4 and IL-17A. The immunosuppressive activity of nicotine is mediated primarily through the alpha-7-nicotinic acetylcholine receptor, which mediates signaling pathways which inhibit the activation of NF-κB, and incudes phosphorylated STAT3-dimers which inhibit transcription of pro-inflammatory cytokines^51^. While we have previously shown that acute morphine administration induces the activation of NF-κB^52^, very little is known regarding the regulation of gene expression in immune cells following chronic administration of either morphine or nicotine (or smoke), or the combination of the two. There is some evidence that the activation of nicotinic acetylcholine receptors induces the release of endogenous opioid peptides, including the enkephalins and β-endorphin^53^, and perhaps the combination of chronic morphine together with endogenous opioid agonist potentiates the level of opioid and is responsible for the suppression of cytokine expression we observed here.

We addressed the status of microglial cell activation, and we found that HIV infection, in the absence of smoke or morphine, induce minimal microglial activation. This is in contrast to clinical studies which demonstrate a significant association between microglial activation and HIV infection^54^. In addition, we observed that the combination of chronic morphine exposure and HIV also failed to induce significant enhancement of microglial activation, in contrast to studies which report that the combination of these insults induce activation^54–56^. In contrast, we observed significant augmentation of microglial reaction with the combination of smoke and HIV, and even more substantial activation with the combination of smoke, morphine, and HIV. This result was somewhat surprising given the absence of induction of most pro-inflammatory cytokines in blood in these experimental groups. Moreover, we found the combination of smoke and chronic morphine, in the absence of HIV infection, significantly enhanced microglial activation. We are not aware of any studies which deal with the capacity of the combination of tobacco smoke and chronic morphine to modulate inflammation. However, both of these insults may be related to their ability to impair the integrity of the blood–brain barrier^57–59^. Our results are consistent with the notion that the combination of these two insults provides a potent neuroinflammatory stimulus, leading to neuro-cognitive decline in HIV-infected patients who are smokers^60^. Based on these results, these insults would be expected to promote neurodegeneration among individuals who are infected with HIV, and who have a history of both tobacco smoke and chronic opioid administration.

In conclusion, we have found that both smoke and chronic morphine administration alter the inflammatory response in a humanized mouse model system. In addition, the combination of smoke and morphine treatments exhibit unique pro-inflammatory activity at the level of microglial cell activation both in the HIV-infected, and uninfected animal groups. This is the first study that we are aware of that examine the combination of smoke and chronic morphine administration on the neuroinflammatory response to HIV. The combination of smoke exposure, and chronic administration of an opiate has clinical significance given the frequent coincidence of tobacco and opiate use among the intravenous drug abuse population that is also HIV-infected. We believe these results suggest that the combination of tobacco and opiate insults is likely to contribute to the neurodegeneration associated with HIV infection.

## Supplementary information

Supplementary Information.
