# Supplementary material for: Tobacco smoke and morphine alter peripheral and CNS inflammation following HIV infection in a humanized mouse model
Source: Sci Rep. 2020 Aug 19;10:13977. doi: 10.1038/s41598-020-70374-7 (PMC7438518; doi:10.1038/s41598-020-70374-7)
Supplement: Supplementary file 1 — Supplementary Information. [file 41598_2020_70374_MOESM1_ESM.pdf]

Supplemental Material for:

Tobacco smoke and morphine alter peripheral and CNS inflammation following HIV infection in a humanized mouse model.

William D. Cornwell[*1], Uma Sriram[*2], Alecia Seliga[2], Viviana Zuluaga-Ramirez[2], Sachin Gajghate[2], Slava Rom[2,3], Malika Winfield[2], Nathan A. Heldt[2,3], David Ambrose[1], Thomas J. Rogers[1,3] and Yuri Persidsky[2,3].

[1]Center for Inflammation, Translational and Clinical Lung Research and [2]Department of Pathology and Laboratory Medicine [3]Center for Substance Abuse Research, Lewis Katz School of Medicine, Temple University, Philadelphia, PA 19140, USA.

Supplemental Table 1. Antibodies used for Flow Cytometry

Antibody	Clone	Flurochrome	Company
CD45	HI30	700	BD Biosciences
CD3	UCHT1	BV510	BioLegend
CD4	RPA-T4	APC	eBioscience
CD8	RPA-T8	eFlour450	eBioscience
CD20	2H7	BUV395	BD Biosciences
CD56	NCAM16.2	BUV395	BD Biosciences
PD1	EH12.2H7	perCP-Cy5.5	BioLegend
Perforin	dG9	PeCy7	BioLegend
Granzyme B	CLB-GB11	FITC	BioLegend

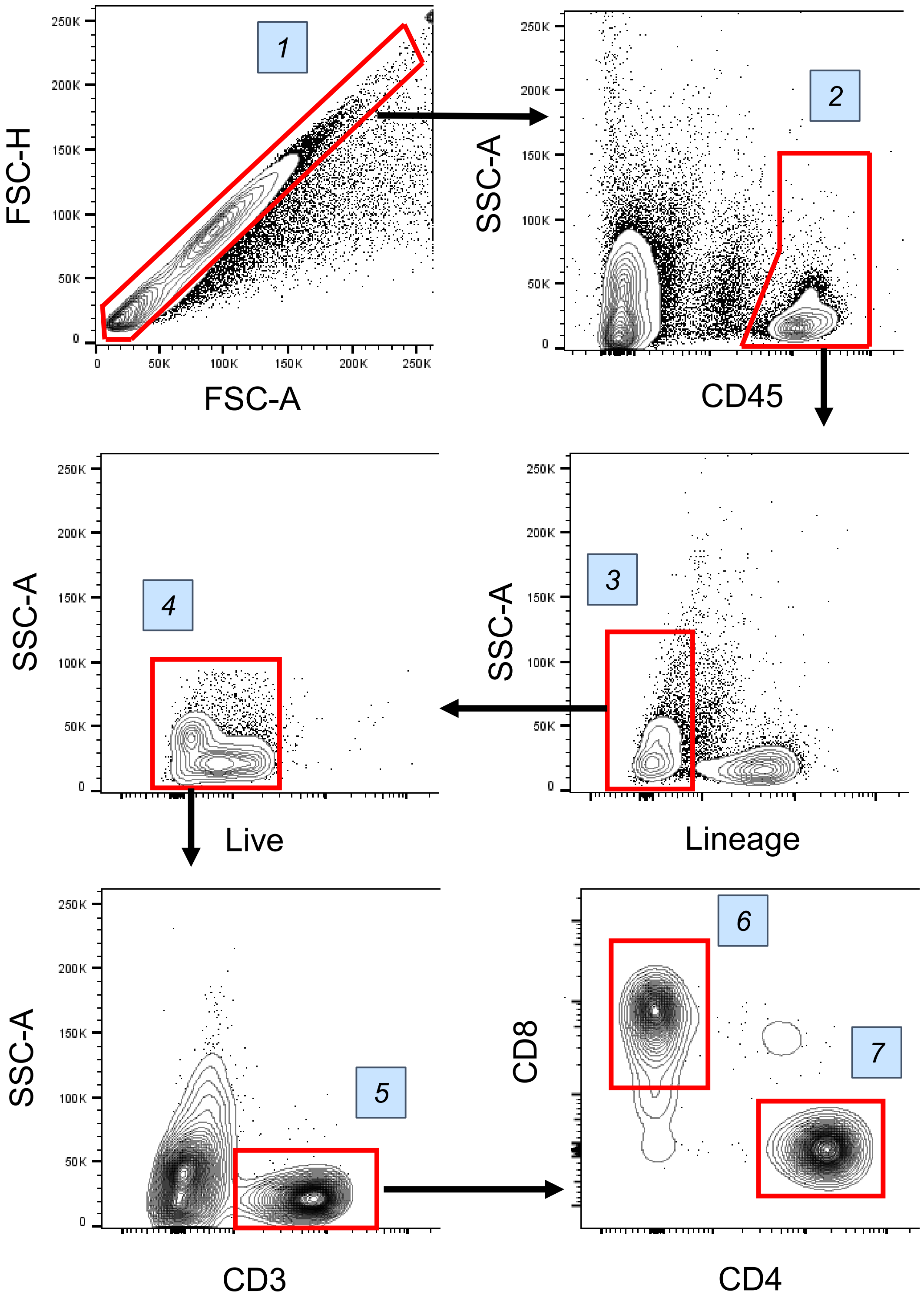

Supplemental Figure 1. Gating strategy for flow cytometry. First, single cells were Isolated (step 1), followed by gating for human CD45-pos leukocytes. After removal of non-T cells and dead cells (steps 3 and 4), the CD3 cells were gated (step 5), and then the individual CD8 (step 6) and CD4 cells were gated (step 7).

	Control vs Smoke	Control vs Smoke + Morphine
hIL-10	3.38	4.51
hTNFa	2.54	3.96
hIL-2	2.53	2.85
CXCL8	2.32	9.01
CCL22	1.65	2.07
CCL2	1.45	3.92
hIL-6	0.96	4.72
hIL-12p70	0.96	0.25
hIL-1b	0.77	6.64
hIL-4	0.72	0.75
hIL-13	0.66	1.47
hIL-16	0.58	-0.04
CCL13	0.54	0.64
hIL12/23p10	0.44	1.31
hVEGF	0.36	0.37
hIL-17A	0.21	1.11
CCL4	0.02	1.35
hIL-7	-0.18	-0.70
hIFNg	-0.33	3.85
hIL-15	-0.86	-0.73
hIL-1a	-0.88	-0.96
hTNFb	-1.09	-1.08
CCL17	-1.29	-0.32
hIL-5	-1.51	-1.15
hGM-CSF	-1.59	0.87
CCL3	-3.76	-1.97
CCL11	-4.52	-4.42
CXCL10	-4.85	-1.41
CCL26	-6.25	-6.30

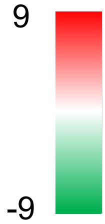

Supplemental Figure 2. Heat map comparing the level of protein expression in cells from control mice with: (left column) cells from mice subjected to smoke; or (right column) cells from animals given both morphine and smoke (right column).

	HIV vs Smoke+ HIV	HIV vs Morphine+ HIV	HIV vs Smoke+ Morphine+ HIV
hIL-1b	1.43	2.28	0.13
CXCL10	1.22	0.97	0.83
hIL-10	1.03	1.27	0.72
hIL-2	0.96	1.03	0.53
CCL17	0.79	0.61	0.47
hTNFa	0.67	0.69	-0.02
CCL2	0.49	0.43	-0.41
CCL22	0.47	0.92	0.41
hIFNg	0.46	0.65	-0.89
CXCL8	0.45	1.08	2.75
hIL-4	0.42	0.34	-2.06
hIL-12p70	0.37	0.42	0.14
CCL11	0.23	0.38	0.15
hIL-6	0.19	0.84	-0.24
hGM-CSF	0.15	0.84	-1.54
CCL4	0.15	0.57	0.15
CCL3	0.11	0.38	-0.16
hIL-16	0.05	0.06	-0.34
CCL13	0.02	-0.38	-0.45
hTNFb	0.01	-0.02	-0.11
hVEGF	-0.02	0.03	-0.39
hIL-15	-0.04	-0.02	-0.51
hIL-13	-0.04	-0.13	-0.57
hIL-7	-0.09	0.32	-0.44
hIL-1a	-0.26	0.10	-0.56
hIL12/23p10	-0.72	0.54	-1.03
hIL-5	-0.74	-0.17	-0.17
CCL26	-0.87	-0.40	-0.69
hIL-17A	-1.17	-0.42	-1.79

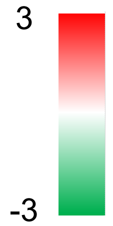

Supplemental Figure 3. Heat map comparing the level of protein expression in cells from HIV-infected mice with: (left column) cells from HIV-infected mice subjected to smoke; or (middle column) cells from HIV-infected animals given morphine; or (right column) cells from HIV-infected animals given both morphine and smoke.

	Control vs HIV	Smoke vs Smoke+ HIV	Smoke+ Morphine vs Smoke+ Morphine+ HIV
CCL2	3.86	2.90	-0.47
hIFNg	2.96	3.76	-1.78
hIL-10	2.71	0.37	-1.08
CXCL8	2.48	0.62	-3.78
hTNFa	2.26	0.39	-1.72
CCL13	1.20	0.68	0.11
hIL-2	1.01	-0.57	-1.31
hIL-4	0.76	0.46	-2.04
hIL-13	0.76	0.06	-1.28
hIL-6	0.73	-0.04	-4.23
hIL12/23p10	0.55	-0.61	-1.80
hVEGF	0.37	-0.01	-0.39
CCL22	0.18	-0.99	-1.47
hIL-1b	0.16	0.82	-6.36
hIL-17A	0.05	-1.34	-2.86
CCL4	0.00	0.13	-1.19
hGM-CSF	-0.17	1.56	-2.58
hIL-12p70	-0.27	-0.85	-0.38
hIL-15	-0.31	0.51	-0.10
hIL-16	-0.87	-1.41	-1.17
hIL-1a	-0.91	-0.29	-0.51
hIL-7	-0.96	-0.86	-0.70
hTNFb	-1.52	-0.41	-0.55
CXCL10	-1.87	4.21	0.38
hIL-5	-2.86	-2.10	-1.88
CCL17	-3.19	-1.10	-2.39
CCL3	-3.34	0.53	-1.54
CCL11	-4.37	0.39	0.20
CCL26	-5.69	-0.30	-0.09

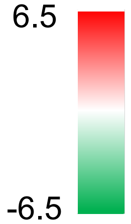

Supplemental Figure 4. Heat map comparing the level of protein expression in: (left column) cells from control animals with cells from HIV-infected animals; or (middle column) cells from smoke-treated animals with cells from HIV-infected smoke treated animals; or (right column) cells from mice treated with both smoke and morphine with cells from HIV-infected mice treated with both smoke and morphine.
